# Dependence of contrast to noise ratio between ablation scar and other tissues on patient heart rate and flip angle for late gadolinium enhancement imaging of the left atrium

**DOI:** 10.1186/1532-429X-14-S1-O107

**Published:** 2012-02-01

**Authors:** Sathya Vijayakumar, Eugene Kholmovski, Christopher McGann, Nassir F Marrouche

**Affiliations:** 1 UCAIR, Dept. of Radiology, University of Utah, Salt Lake City, UT, USA; 2 CARMA Center, University of Utah, Salt Lake City, UT, USA; 3 Dept. of Cardiology, University of Utah, Salt Lake City, UT, USA

## Background

Radiofrequency (RF) ablation of the left atrium (LA) and pulmonary vein ostia has become a clinically acceptable therapy for atrial fibrillation (AF). High-resolution 3D Late Gadolinium Enhancement (LGE) imaging can detect pre-ablation re-modeling of the LA wall and also visualize post-ablation scar in AF patients treated using RF ablation. The 3D Inversion Recovery prepared Gradient Recalled Echo (IR-GRE) LGE sequence typically used, is dependent on parameters like the flip angle and inversion time. However the effect of patient heart rate (HR) on the CNR between scar and fat, myocardium and blood has not been studied before. Here, we simulate the IR-GRE sequence acquisition at different HRs with the aim of improving the performance of this approach to assess post ablation scars.

## Methods

In 3D LGE of the LA, the inversion pulse is applied every heartbeat (RR interval) after which data are acquired. Realistic T1 values at 3T for post-ablation scar, myocardium, fat and blood were estimated from analyzing TI-scout images of 50 patient studies to be 120, 420, 254 and 312 ms respectively. Simulations were performed with these values of T1 and different HRs, TR=3.1 ms, TE=1.4 ms, and multiple flip angles, using MATLAB (Mathworks Inc. Natick, MA).

Retrospective analysis of 58 patients' data acquired on a 3T Verio scanner (Siemens Healthcare, Germany) was performed. High resolution LGE images were acquired 20 minutes after contrast injection (0.1 mmol/kg, Multihance (Bracco Diagnostics, NJ)) using a 3D respiratory navigated, IR-GRE pulse sequence with TR/TE=3.1/1.4 ms, flip angle 13°, FOV=400x400x100 mm and voxel size=1.25x1.25x2.5 mm. Measurements of normal myocardium, blood pool and scar region (mean and standard deviation) were made in the LGE images using Osirix software in 58 patients with different HRs (3 or 6 months post ablation) by drawing regions of interest in the respective tissue type. CNR was computed as the difference in signal intensity between scar and other tissues, divided by the standard deviation of the blood pool signal.

## Results

**Figure 1:**
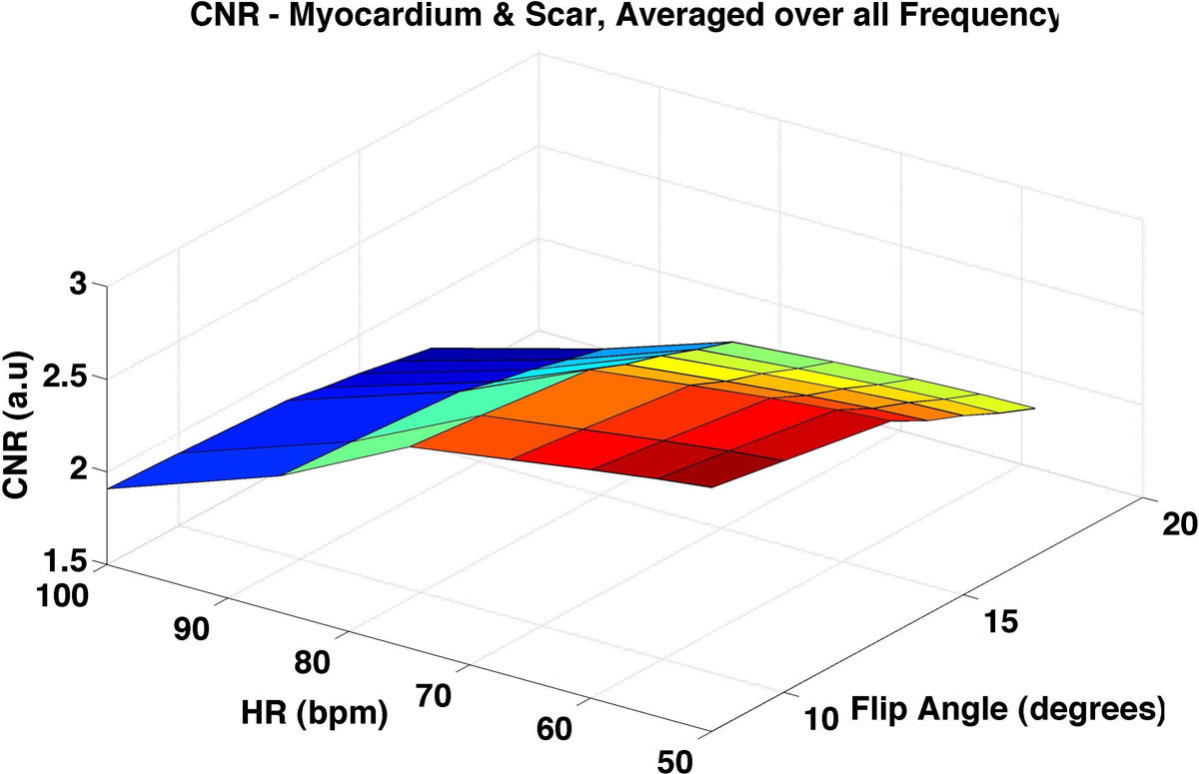
Simulations results showing the dependence of CNR between mycardium and scar on HR and flip angle.

**Figure 2:**
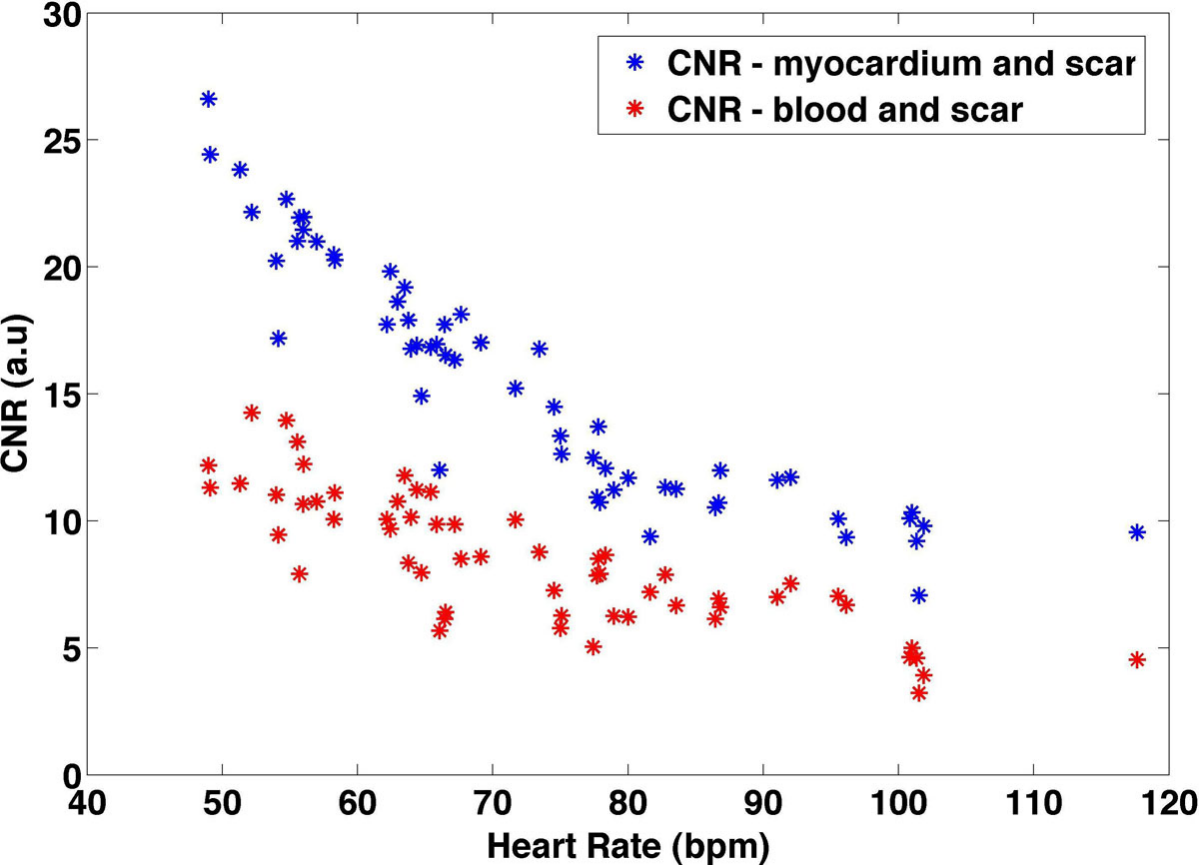
Results of CNR between myocardium and scar and blood and scar as measured from patient data.

Figure 1 shows the CNR between myocardium and scar obtained from the simulations. Note that the CNR between scar and other tissues decreases with increasing HR. Also, the use of a smaller flip angle in patients with a high HR would yield better CNR between scar and tissue. Figure 2 shows the results of the computed CNRs from the patient dataset. CNRs between normal myocardium and scar and blood and scar are shown as a function of HR. Note that the results based on patient data follow the simulated curves. With a lower HR, the CNR between blood and scar and myocardium and scar is higher.

## Conclusions

From the simulations and the patients’ images, we see that the CNR between scar and myocardium and scar and blood depends on HR and may be improved if lower flip angles are used for higher HR. This dependence of CNR on HR may also impact automated segmentation algorithms, if the HR is included in the algorithm.

## Funding

None.

